# Effects of Hunger on Visual Perception in Binocular Rivalry

**DOI:** 10.3389/fpsyg.2019.00418

**Published:** 2019-03-12

**Authors:** Xin Weng, Qi Lin, Ye Ma, Yu Peng, Yang Hu, Ke Zhou, Fengtao Shen, Huimin Wang, Zhaoxin Wang

**Affiliations:** ^1^ Key Laboratory of Brain Functional Genomics (MOE and STCSM), Institute of Cognitive Neuroscience, School of Psychology and Cognitive Science, East China Normal University, Shanghai, China; ^2^ Shanghai Changning-ECNU Mental Health Center, Shanghai, China; ^3^ Graduate School of Beijing Normal University (Zhuhai), Zhuhai, China; ^4^ NYU-ECNU Institute of Brain and Cognitive Science, New York University Shanghai, Shanghai, China

**Keywords:** binocular rivalry, hunger, dominance time, b-CFS, probe-detection

## Abstract

The effect of hunger on visual perception is largely absent from contemporary vision science. Using a well-established visual phenomenon termed binocular rivalry, this study was carried out to investigate the effects of hunger on visual perception. A within-subject design was applied in which participants attended two sessions before and after their lunch or dinner, i.e., a hunger state and a satiated state. In Experiment 1, we found that the mean dominance times to food-related pictures were larger in the hungry condition than that in the satiated condition, while the mean dominance time to the non-food stimuli were unaffected. In Experiment 2, we found the times to break through continuous flash suppression (b-CFS) for both food-related and non-food-related pictures were not affected by hunger. In Experiment 3, a probe-detection task was conducted to address possible response-biases. Our findings provide evidence that hunger biases the dynamic process of binocular rivalry to unsuppressed and visible food stimuli, while processing suppressed and invisible food-related was unaffected. Our results support the notion that the top-down modulation by hunger on food-related visual perception is limited to visible stimuli.

## Introduction

Recent studies suggest that visual perception can be modulated by multiple top-down factors, such as affection (Yang et al., 2007; Tsuchiya et al., 2009; Gray et al., 2013), even cross-modal integration (Zhou et al., 2010, 2012). Hunger is a complex behavioral state that elicits intense food seeking and consumption (Atasoy et al., 2012), hence provides a strong top-down motivation for food. However, the effect of hunger on visual perception is largely absent from contemporary vision science (Firestone and Scholl, 2016). Note that the modulation effects of different top-down factors on visual perception may be different. For example, it was reported that some classes of stimuli may be processed unconsciously, such as fearful faces (Yang et al., 2007), while the effect of happy faces on subliminal visual perception is limited (Yang et al., 2007; Tsuchiya et al., 2009). Thus, investigating the effects of hunger on visual perception could provide further insights on possible dynamic top-down modulations on visual perception and how the visual system works.

It is widely reported that hungry modulates visual perception with clearly visible food-related stimuli. For example, hunger participants tend to interpret ambiguous stimuli as food (Lazarus et al., 1953). At the neural level, studies also revealed that food cues increase dopamine levels in the striatum and that these increases are correlated with hunger and craving (Volkow et al., 2002), hunger selectively modulates dopamine levels and corticolimbic activation to food stimuli (LaBar et al., 2001; Siep et al., 2009), even to food-related odor (Bragulat et al., 2010). However, it is still unclear whether hunger modulates visual processing of invisible or unconscious stimuli, conflicting findings had been reported. To our knowledge, only two studies have focused on the effects of hunger on the invisible or unconscious processes of food-related stimuli, and the results are inconsistent. Radel and Clément-Guillotin reported that the processing of food-related words can be modulated by degree of motivation for food unconsciously on a semantic level (Radel and Clément-Guillotin, 2012). In their study, after a 67 ms premask, a word appeared for 33 ms and was followed by a 33 ms blank screen and a 67 ms postmask. On the contrary, a hunger-related bias was only found in selective attention but not in unconscious processes when words were shown very quickly and masked (Mogg et al., 1998). Clearly, further study was needed to address the question of possible modulation effects of hunger on subliminal and suprathreshold visual perception.

Binocular rivalry (Wheatstone, 1838) is attained when different stimuli are presented to each eye, resulting in perceptual switches between the two stimuli. Since the stimulus remains unchanged while perception alternates between two alternatives, binocular rivalry is an ideal paradigm to test direct effects on perception (Alais and Blake, 2005). Imaging studies revealed that brain regions, such as the fusiform face area and parahippocampal place area (Tong et al., 1998), even the lateral geniculate nucleus (Wunderlich et al., 2005), can be selectively activated by alternative perception. It was found that binocular rivalry can be affected by physical factors, such as contrast and luminance (Levelt, 1965; Brascamp et al., 2015). Interestingly, images with affective value — such as reward (Marx and Einhauser, 2015), and even gossip (Anderson et al., 2011), — dominate in binocularly rivalry over affectively neutral images, supposedly by top-down modulation on visual perception. Moreover, the binocular rivalry paradigm may be used to investigate unconscious processing. Levelt proposed a term of stimulus strength, which is the degree to which the physical characteristics, such as brightness, of one eye’s stimulus enable that stimulus to perceptually suppress the stimulus presented to the other eye (Levelt, 1965; Brascamp et al., 2015). Note that increase of the stimulus strength in one eye will only reduce the average perceptual dominance duration of the other eye’s stimulus, which could be explained as the ability of the unconscious stimuli to break suppression. As hungry people have a bias to overrate the brightness of pictures of food (Gilchrist and Nesberg, 1952), this effect may be detected if the stimulus strength of food can be modulated by hunger.

A more optimal technique for examining preconscious processing using binocular rivalry was introduced by Tsuchiya and Koch, termed continuous flash suppression (CFS) (Tsuchiya and Koch, 2005). In the case of CFS, a stimulus is prevented from reaching awareness by presenting strong dynamic noise to the opposing eye, allowing for long durations of suppression (Tsuchiya and Koch, 2005). Thus, it provides stronger suppression than is typically seen with standard (static) binocular rivalry designs, and the time stimuli need to overcome CFS and emerge into awareness (breaking CFS or b-CFS) could be an index of their potency to gain access to awareness (Stein et al., 2011). In the first groundbreaking experiments using b-CFS, Jiang et al. (2007) found high-level stimulus properties, such as upright face (vs. inverted face) can remain effective during interocular suppression (Jiang et al., 2007), reflecting preserved higher-level processing differences under rivalry suppression. It was also found that various classes of stimuli, such as fear, may break the continuous flash suppression (b-CFS) differently from neutral, happy, or angry faces [for a review, see Gayet et al., 2014], indicating that the b-CFS task can be used to study top-down modulations on unconscious processing.

In the present study, we used a binocular rivalry paradigm and the b-CFS task to investigate the effects of hunger on food-related visual perception. It is believed that pictorial stimuli are more representative of the world than words and contribute to a more accurate assessment of automatic processing than words that initially involve lexical processes (Bruce and Jones, 2004; Johansson et al., 2005; Papies et al., 2009), food pictures can also lead to attentional bias (Fadardi and Bazzaz, 2011). Therefore, we used pictures as stimuli in the present study. In light of previous findings that images with affective value tend to dominate in binocular rivalry over affectively neutral images, and some affective stimuli (such as fearful faces) were more likely to break CFS earlier than neutral images, as hunger is a strong motivation for food, we hypothesized that in the hungry condition, the dominance time of food-related stimuli should be longer than that of non-food related stimuli, and food-related stimuli should break through CFS earlier than non-food related stimuli.

## Materials and Methods

### Participants

Twenty one Chinese students from East China Normal University (ECNU) (9 males; age 22–28, mean age = 24.1, SD = 1.8) voluntarily attended the current study. All were right-handed with normal or corrected-to-normal vision and normal color perception, and reported no psychiatric or neurological history. After completing all tasks, participants were debriefed and paid as compensation for their time. Written informed consent was obtained from all participants and the present study was approved by a local ethics committee.

### Stimuli

**Figure 1:**
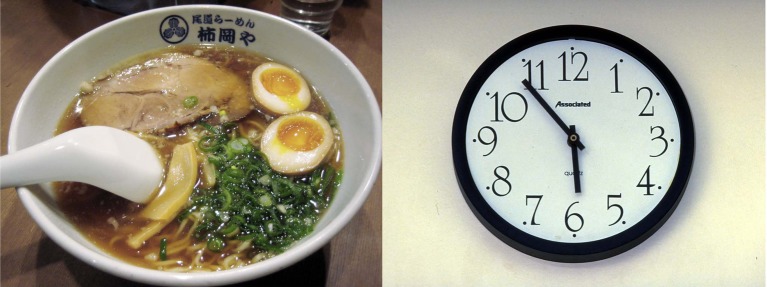
A pair of sample stimuli. Left, food; right, non-food.

Eight pairs of pictures were chosen from the International Affective Picture System (IAPS) (Lang et al., 1999), half of them were food, see Figure 1 as one example pair. The valence, arousal, and luminance were matched between food and non-food stimuli. They were displayed on a 17-in. standard screen LCD monitor, which was set to 1,280 × 1,024 pixels and 100 Hz temporal resolution, and presented dichoptically using a haploscopic mirror system attached to a head-and-chin rest. Each image subtended 3.0° × 2.5° of visual angle and the viewing distance was 85 cm.

### Experiment 1

Experiment 1 tested the hypothesis that the dominance times of food images during binocular rivalry are longer in the hunger condition than that in the satiated condition when they are consciously aware of the images. And the suppression times of food images should be shortened by hunger due to unconscious processing of food images.

### Procedure

Each participant underwent two behavioral sessions (hungry and satiated). All participants were told to complete the task twice to ensure the stability of the data. The participants attended this experiment before and after lunch/dinner. The participants attended the first session at about 11:30/17:30 before meals. Note that typically the lunch/dinner time was set to start at about 11:00/17:00 locally. Before the experimental session, each participant would be first trained to adapt to the experimental procedure with two pictures that were not used later. They would be asked to rate on a seven point Likert scale ranging from 0 (not at all) to 6 (completely) on the following terms based on subjective feeling: happiness, tiredness, hungriness, enthusiasm, and impatience. After that they were instructed to come back after 1 h to finish the second session and were told that they could take their meals in the interim.

Each experimental session comprised of 16 trials. Participants pressed the space bar of the computer keyboard to trigger the presentation of a pair of two binocular rivalry stimuli on the computer screen for 60 s. The participants task was to track his/her binocular rivalry perception by pressing “1” when they saw one image, pressing “2” when it switched to another one and pressing “3” when both the images could be seen. At the end of a 60 s trial, the participants would be asked to rest for 30 s with their eyes closed. Then they began the next trail. There was a 3-min rest after eight trials.

Participants’ level of subjective feeling of hunger was higher in the hungry condition (*M* = 4.0, SD = 1.7) than that in the satiated condition (*M* = 0.7, SD = 1.2), *t* = 7.6, *p* < 0.001. No significant difference was found in other subjective terms, *t*s < 1.3, *p*s > 0.1.

### Experiment 2

In the experiment 2, we further tested the hypothesis that the suppression times of the food images can be modulated by hunger. A b-CFS paradigm was adopted. We hypothesized that food images can break through the CFS faster than non-food images.

### Procedures

**Figure 2:**
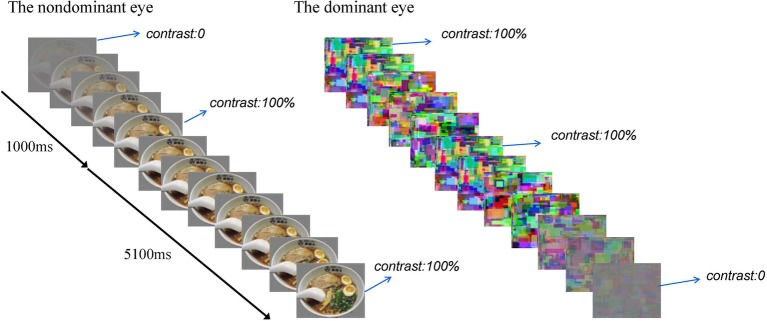
A brief overview of a b-CFS trial. The contrast of the test figure was ramped up gradually from 0 to full contrast within 1,000 s, and then remained constant until the subjects made a button-press response, the contrast of the dynamic noise was ramped down gradually from full contrast to 0 within 5,100 ms after the test figure reached its full contrast.

Each experimental session comprised eight blocks. Each block consisted of 16 b-CFS trials. The food images were used in half of the trials and the non-food images in the other half. These images were presented randomly. At the beginning of each trial, dynamic noise patterns (Mondrain patterns) were presented to the subjects’ dominant eye at full contrast, and the test figure was presented to the subjects’ non-dominant eye at the region corresponding to the location of the noise pattern. The contrast of the test figure was ramped up gradually from 0 to full contrast within 1,000 ms starting from the beginning of the trial, and then remaining constant until the subjects made a button-press response to indicate whether they saw the food image or the control image (Zhou et al., 2010), the contrast of the dynamic noise was ramped down gradually from full contrast to 0 at a rate of 2% every 20 ms within 5,100 ms, starting from 1,000 ms after the test figure reached its full contrast (Yang et al., 2007). The rate of stimuli presentation was 20 ms per image. At the end of each block, the participants would be asked to rest for 30 s with their eyes closed. Then they began the next block. There were 6 practice trials using different pictures and 128 experimental trials. See Figure 2 for a brief overview.

## Data Analysis

Repeated measures ANOVA and paired *t*-test were used for statistical analysis.

## Results

### Experiment 1

**Figure 3:**
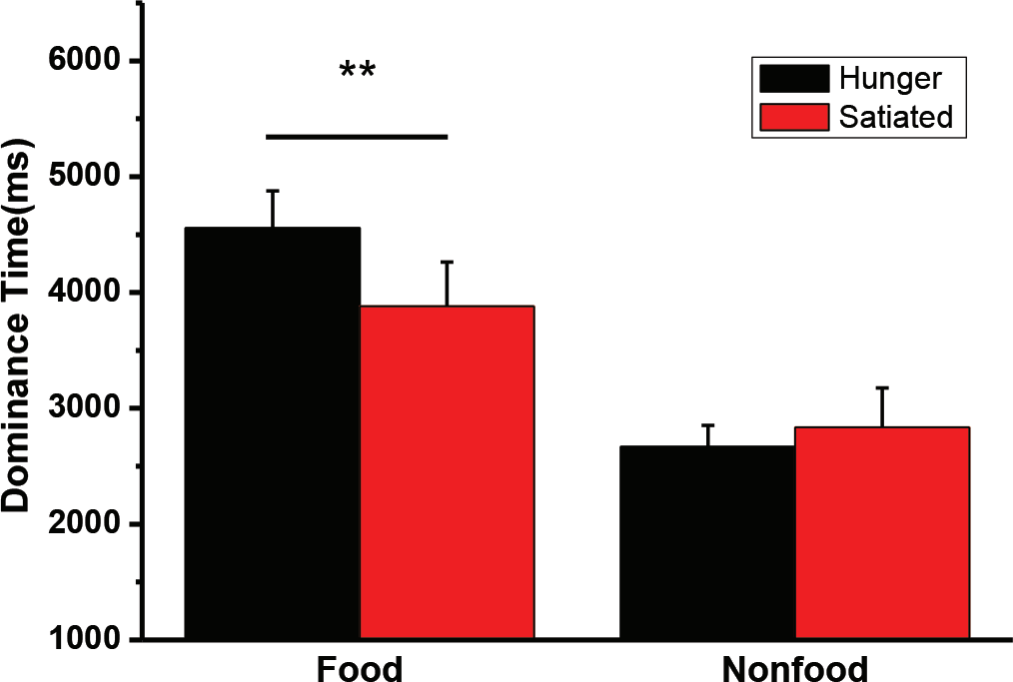
The mean duration time (and SEs) for dominant perceptions of food and nonfood stimuli in the hungry and satiated states. **p = 0.004.

A 2 state (hunger status: hungry vs. satiated) × 2 type (visual image: food vs. non-food) repeated measures ANOVA was conducted on the mean dominance time. The interaction was significant, *F*(1, 20) = 11.5, *p* = 0.003. A main effect of stimuli was also found, *F*(1, 20) = 36.2, *p* < 0.001. Paired *t*-test was performed to further compare the dominance for between the motivation status (hungry vs. satiated). Hunger did not affect the dominance time of the non-food images *t*(20) = −0.8, *p* = 0.44, while participants had a longer dominance time on the food images in the hungry state (*M* = 4,556 ms, SD = 1,468 ms) than in the satiated state (*M* = 3,880 ms, SD = 1,753 ms), *t*(20) = 3.2, *p* = 0.004. Individual mean dominance time was illustrated in Figure 3.

### Experiment 2

**Figure 4:**
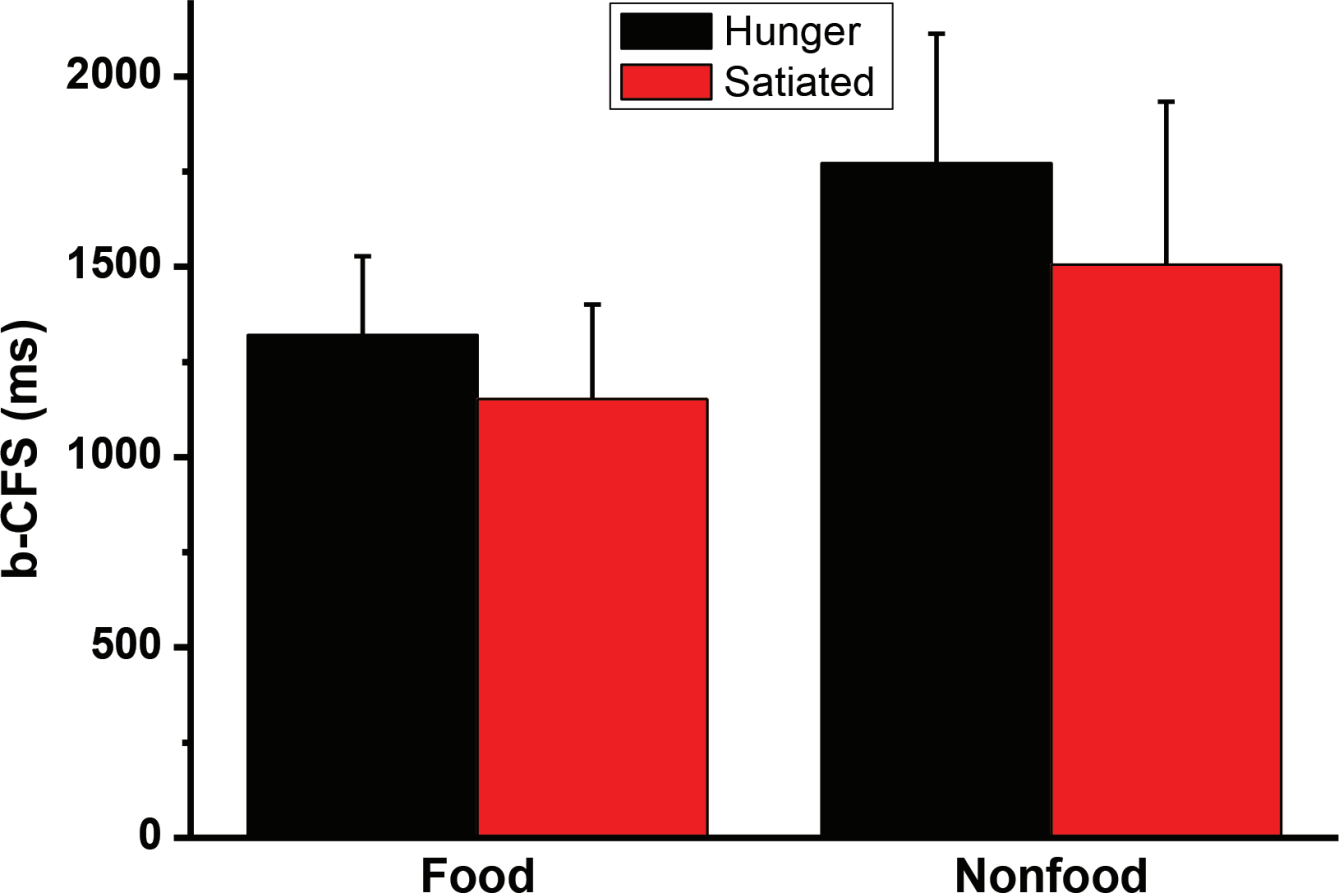
The times to break through Continuous Flash Suppression (b-CFS). Error bars represent standard deviations.

A 2 (motivational status: hungry vs. satiated) × 2 (visual images: food vs. control) repeated measures ANOVA analysis revealed that the interaction was not significant, *F*(1, 20) = 2.2, *p* = 0.15. The main effect of stimulus type was significant, *F*(1, 20) = 73.0, *p* < 0.001. The main effect of session was also significant *F*(1, 20) = 10.2, *p* < 0.004. Paired sample *t*-test was performed to further compare the b-CFS for food image and control image. It was found that hunger did affect the b-CFS of the food images, *t*(20) = 3.2, *p* = 0.005, and the control images, *t*(20) = 2.9, *p* = 0.009. See Figure 4. To address between-subject variability we also used a simple latency-normalization method (Δ*RT*_NORMALIZED_ = 100 ∗(*RT_A_* − *RT_B_)/RT_OVERALL_*), and a log-transformation method (Gayet and Stein, 2017) to transform the data and reanalyze the data, no significant interaction was found, *p*s > 0.29.

### Experiment 3

Although several approaches were used to conceal the purpose of the study, the results reported here (effect in rivalry but not in b-CFS) can be explained by rivalry being much more susceptible to response biases as the purpose of the study is relatively clear. A third experiment was designed to include a more objective measure, i.e., a probe detection paradigm that allow for recording dominance times indirectly through the detection of a probe presented to one eye (Wales and Fox, 1970; Alpers and Gerdes, 2007). We presented visual probes, a small circle, embedded in either the food or the nonfood images. If one kind of picture is suppressed more often than another less probes should be detected in it. Of interest, if a probe is detected, it is impossible to know whether the dot appeared in the food or in the nonfood stimuli. This further eliminates response bias. Thus, the objective count of detected probes would be an objective index of binocular rivalry.

## Materials and Methods

### Participants

Thirty two Chinese students from East China Normal University (ECNU) (4 males; age 18–28, mean age = 22.2, SD = 2.1) voluntarily attended the current study. All were right-handed with normal or corrected-to-normal vision, normal color perception, and reported no psychiatric or neurological history. After completing all tasks, participants were debriefed and paid as compensation for their time. Written informed consent was obtained from all participants and the present study was approved by a local ethics committee.

### Stimuli

As in Experiment 1, the eight pairs of pictures and the same apparatus were used. A very small circle probe with black and white sine-wave gratings (4.5 cycles, begins and ends with black lines, 45 pixels, about 0.25° visual angle) appeared in the center of the stimuli, and disappeared in 200 ms (Nguyen et al., 2001). This probe appeared randomly in either eye 1–1.5 s after the participants indicated that one picture dominated. The ratio of probes appearing on the food or the non-food stimuli was equal. This experiment was controlled and responses were recorded by Matlab 2014a (MathWorks Inc, USA) and Psychtoolbox-3.0.11 (http://psychtoolbox.org/).

### Procedure

The procedure was very similar to that of Experiment 1. Each participant underwent two behavioral sessions (hungry and satiated). All participants were told to complete the task twice to ensure the stability of the data. The participants attended this experiment before/after lunch or after lunch/before dinner. For the before/after lunch group, the participants attended the first session at about 11:30, before meals. Note that typically the lunch/dinner time was set to start at about 11:00/17:00 locally. Before the experimental session, each participant would be first trained to adapt to the experimental procedure with two pictures that were not used in later test. They would be asked to rate on a seven point Likert scale ranging from 0 (not at all) to 6 (completely) the following terms based on subjective feeling: happiness, tiredness, hungriness, enthusiasm, and impatience. Then they were given a meal (total calories 3,134 kj) to eat. After about 90 min, they were instructed to finish the second session. For the after lunch/before dinner group, the participants first consumed a meal as lunch provided by the experimenter, and then finished the first session. They were instructed to come back for the second session at about 16:30/17:30 without dinner. The timing of these two sessions was carefully selected in a natural way and counterbalanced among participants.

Participants were asked to code any change in their percept and to respond probes in the pictures simultaneously. There were several practice trials, in which a different set of food/nonfood stimuli was used. First, the coding of the percepts (food vs. nonfood) with three different keys with their right hands, one for the one stimuli two for the other stimuli (determined by instruction), and three for mixed perception. Then a 200 ms probe was presented randomly in approximately 1,000–1,500 ms, and the location was pseudorandomly assigned. Participants was asked to press “q” with their left hand if they saw the probe. If the percept was not changed, another probe was then presented after 1–1.5 s with pseudorandomly assigned location. At the end of a 60 s trial, the participant would be asked to rest for 30 s with their eyes closed. Then they began the next trail. There was a 3 min rest after eight trials.

### Data Analyses

To minimize the probability of false-positives, probes were only counted as detected if the key presses were subsequent reactions to presented probes (Alpers and Gerdes, 2007). Repeated measures ANOVA were used.

## Results

**Figure 5:**
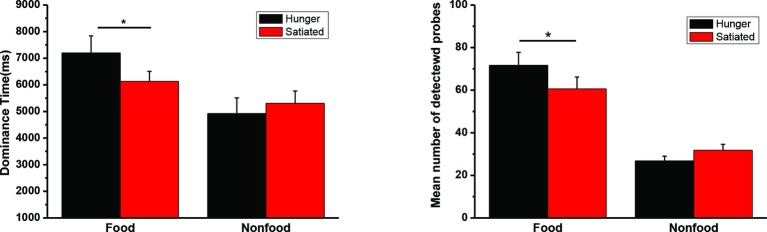
The mean duration time (and SEs) for dominant percepts of food and nonfood stimuli in Experiment 3 (left panel). The mean number of detected probes (and SEs) appearing in the food or nonfood stimuli (right panel). *p < 0.05.

The mean duration time for dominant percepts of food and nonfood stimuli and the mean number of detected probes in Experiment 3 were illustrated in Figure 5. The interaction was significant for both dominance time, *F*(1, 34) = 6.49, *p* = 0.016, *η*^2^ = 0.160, and detection rate, *F*(1, 34) = 6.14, *p* = 0.018, *η*^2^ = 0.153. We also found a significant main effect of food/nonfood, *F*s(1, 34) > 21.8, *p*s < 0.001, *η*^2^ > 0.391, but not of session, *F*s(1, 34) < 0.8, *p*s > 0.36.

## Discussion

Using binocular rivalry, the results from Experiment 1 revealed that participants had a longer dominance time on the food images in the hungry state than that of the satiated state, while the dominance time of the control images was not affected by hunger. Moreover, Experiment 3 shows that participants’ self-report of what they perceive can be verified by a probe-detection task, indicating our results cannot be explained by possible response biases. The results from Experiment 2 also showed that there were no significant stimuli type (food/non-food) by hunger/satiated states interaction in the b-CFSs, indicating that the potency of food stimuli to overcome CFS to gain access to awareness is comparable to non-food stimuli in both hunger and satiated states. The research reported here provides further evidence that a non-emotional motivational state, such as hunger, is associated with a bias in selective attention to stimuli that are relevant to that motivational state but was only found when the food stimuli were presented in the suprathreshold condition.

The results of Experiment 1 suggest that visual perception to food picture, but not non-food picture, can be modulated by hunger. Consistent with our results, it was also found that fasting resulted in an attentional bias for food stimuli under non-rivaling conditions (Lavy and van den Hout, 1993; Mogg et al., 1998), and subjects who had been deprived of food were more prone to produce food-relevant responses to a series of ambiguous stimuli, and to detect food-related words (Sanford, 1936; Gilchrist and Nesberg, 1952; Radel and Clément-Guillotin, 2012). Chong et al. showed that endogenous attention prolongs dominance durations in binocular rivalry (Chong et al., 2005), while increasing stimulus strength for one eye will not affect the average perceptual dominance duration of that eye’s stimulus, but reduce the average perceptual dominance duration of the other eye’s stimulus (Levelt, 1965; Brascamp et al., 2015). Given that the average perceptual dominance duration of non-food stimuli was not affected, our results can be explained by the top-down endogenous attention modulated by hunger (Piech et al., 2010), while the stimulus strength of the food stimuli was not affected by hunger.

Two lines of evidence indicate that the processing of suppressed food images during binocular rivalry was unaffected by hunger. First, the result of Experiment 1 indicated that the dominance time of the non-food images was unaffected, i.e., the suppression time to food picture was unaffected, thus the stimulus strength of the food images was not modulated by hunger. Second, the results of the Experiment 2 indicated that hunger had no specific effect on the b-CFS of the food images. These results suggest that the hunger modulation on binocular rivalry may not happen at early levels of processing.

One possible reason is that the various levels of cortical organization work together to generate efficient perceptual representations (Grossberg et al., 1997), and different stimuli could be processed at various levels and have different potencies to break through the CFS. For example, faces and words can be processed in specialized brain regions, i.e., the fusiform face area (Kanwisher et al., 1997) and visual word form area (Dehaene et al., 2002) respectively, which may allow early levels of processing of corresponding stimuli. However, until now no evidence of a specialized food area is reported. It is possible that there are too many kinds of food and top-down attention is needed to determine whether a given stimuli is food or not, which limits possible early processing of food-related stimuli. In line with our results, a hunger-related bias was only found in selective attention but not in pre-attentive processes when words were shown very quickly and masked, in which food-related words were shown for 14 ms and masked (Mogg et al., 1998). We also note that a preattentional hunger-related bias was reported in Radel and Clément-Guillotin’s study (Radel and Clément-Guillotin, 2012). In the study a word appeared for 33 ms and was followed by a 33-ms black screen [typically a pattern mask is followed, for example, see Kouider and Dupoux, 2001] and then a 67-ms postmask (Radel and Clément-Guillotin, 2012). As we have found that the effect of a single color rectangle mask is very weak (Wang, 1999), one may argue that their stimuli may be not subconsciously presented and a further comparison between these two paradigms is desired.

We also found main effects of hunger/satiated states in both experiments. Note that for the homogeneity of the procedure, all participants attended the first session of the experiment before their meal and the second session in satiated state, thus the main effects of hunger/satiated states may be due to order effect. Alternatively, these results may also be the reflections of changes in attention by food intake. Although not well studied, attention is arguably modulated by the intensity of hunger [for reviews, see Al-Shawaf, 2016 and Wolraich et al., 1995]. For example, Giles reported in a recent report that sugar intake benefits cognitive processes require sustained attention (Giles et al., 2018) and hunger is associated with an impairment of response inhibition (Loeber et al., 2013), while Ginieis reported that glucose and sucrose ingestion leads to negative cognitive performances including attention (Ginieis et al., 2018). Further study may address this question.

This finding may have one theoretical implication. Firestone and Scholl postulate that vision proceeds without any direct interference from cognition and perception proceeds without any direct, unmediated influence from cognition, or “encapsulation” (Firestone and Scholl, 2016). Here we showed that visual perception to the same food stimuli can be modulated by hunger, evidenced by the selective changes in dominance time to the food stimuli, but no effect was found to the non-food stimuli. These results do not support Firestone and Scholl’s “encapsulation” suggestion. Instead, combined with previous studies, it is becoming obvious that the effects of different kinds of top-down factors on visual perception are different, supporting the notion that perception and cognition are constructed through overlapping and distributed brain networks characterized by top-down activity and context-sensitivity (Hackel et al., 2016).

As far as we know, this is the first study using binocular rivalry to investigate the effects of hunger on visual perception. We provided the first piece of evidence that the dominance time to food image can be modulated by the levels of hunger in binocular rivalry, while the potency of food-related images to break the CFS was not selectively affected by hunger. The study provides further evidence that visual perception can be modulated differently by various top-down factors.

## Ethics Statement

This study was carried out in accordance with the recommendations of the University of Committee on Human Research Protection of East China Normal University with written informed consent from all subjects. All subjects gave written informed consent in accordance with the Declaration of Helsinki. The protocol was approved by the University Committee on Human Research Protection of East China Normal University.

## Author Contributions

ZW and HW designed the study, analyzed the data and wrote the paper. XW, QL, FS, YP, YH, KZ and YM collected data, analyzed the data, and wrote the paper.

### Conflict of Interest Statement

The authors declare that the research was conducted in the absence of any commercial or financial relationships that could be construed as a potential conflict of interest.
